# Emergent Pulmonary Embolectomy and Advanced Glioblastoma Multiforme

**DOI:** 10.1155/2010/862028

**Published:** 2010-12-20

**Authors:** Michael S. Firstenberg, Danielle Blais, Erik Abel, Herbert B. Newton, Juan Crestanello

**Affiliations:** ^1^Division of Cardiothoracic Surgery, The Ohio State University Medical Center, N817 Doan Hall, 410 W 10th Avenue, Columbus, OH 43210, USA; ^2^Departments of Neurology and Neurosurgery, OSUMC, M410-B Starling-Loving Hall, 320 West 10th Avenue, Columbus, OH 43210, USA

## Abstract

Pulmonary emboli are frequent causes of morbidity and mortality in patients with brain tumors. Treatment options are limited in these complex patients. We report a case of successful acute pulmonary embolectomy in a patient with an advanced brain cancer.

## 1. Introduction

Patients with cancers, particularly brain, are at considerable risk for deep vein thrombosis (DVT) and pulmonary embolism (PE). Twenty-six percent of patients with high grade gliomas developed DVTs, and 15% PEs, within 12 months of treatment [1]. Larger and more symptomatic PE's are often treated with thrombolytics, however surgical indications are expanding. Thrombolytics or high-dose anticoagulation for cardiopulmonary bypass is typically contraindicated in patients with significant intracranial pathology [2]. Our favorable surgical management of a patient with a postinfarct ventricular septal defect and acute intracranial hemorrhage [3] has prompted us to consider pulmonary embolectomy to patients with known brain cancers who develop massive PEs.

**Figure 1:**
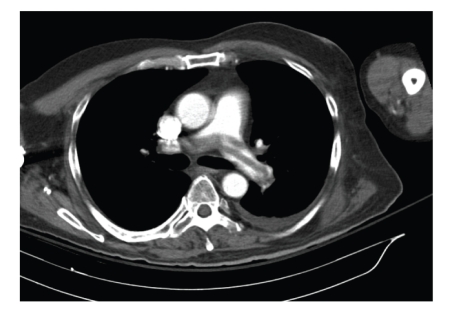
CT pulmonary angiogram showing a large saddle embolus.

**Figure 2:**
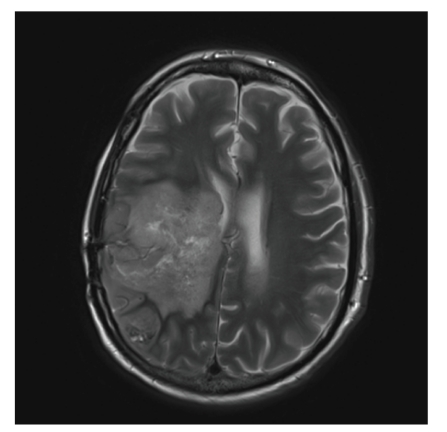
MRI of the brain, 6 weeks after embolectomy, showing stable disease.

Our patient is 47 years old, first diagnosed with a right temporal lobe Glioblastoma multiforme (GBM) in 2005 that was treated with surgical resection and reoperation in 2007 and 2008. He continued to receive cycles of chemotherapy and local irradiation for disease recurrence. He had mild right-sided weakness, short-term memory, and cognitive defects but was functional and living at home. In 02/2009, he developed acute shortness of breath and chest CT confirmed a large saddle pulmonary embolism (Figure 1). He was hemodynamically stable and anticoagulated with a weight-based heparin protocol. Despite adequate anticoagulation, he became fatigued and hypoxemic (O_2_ saturation: 90% on 3 liters via cannula). A transthoracic echocardiogram showed reduced global right ventricular function and his B-type Natriuretic Factor was elevated at 212 pg/mL. His Troponin was 0.14 ng/mL (normal <0.11). His heart rate was 105 bpm and respiratory rate was >20/min and he felt short of breath sitting up and could not lie flat. Because of his brain tumor, based upon established guidelines for the management of PE, thrombolytic therapy was felt to be an absolute contraindication [2]. Because of his anatomically large PE and worsening clinical picture, surgical therapy was offered. Median sternotomy was performed, cardiopulmonary bypass was initiated with full heparinization (goal activated clotting time >350 seconds), the aorta was cross-clamped and the heart was arrested. The main pulmonary artery was opened and large emboli were extracted from the main and left pulmonary artery and lobar branches. The right pulmonary artery was exposed between the superior vena cava and the aorta and addition clots were extracted manually and with a small suction catheter. The pulmonary arteries were irrigated clean with heparinized saline. Total bypass time was 40 minutes and the cross-clamp time was 30 minutes. Immediately postoperatively an IVC filter was placed. He was extubated within 24 hours. His neurologic function was unchanged and postoperative course was unremarkable. Venous duplex scanning showed extensive clot in both legs and systemic anticoagulation with heparin was started 12 hrs postoperatively. He was discharged to a rehabilitation facility, on room air and sodium warfarin, on postoperative day 8. Two weeks later, he returned home and at 6 weeks a repeat MRI showed no disease progression (Figure 2). He survived for 12 additional months at home before returning with an overwhelming infection and septic shock at which time support was withdrawn.

## 2. Discussion

Surgical management of massive PEs is an acceptable therapy. For example, Kadner recently reported a 30-day mortality of 8% following salvage pulmonary embolectomy [4]. Of note, 1 of the 2 deaths was from intracerebral bleeding. In their experience, most patients had significant hemodynamic compromise (32% had a preoperative arrest) and 16% had a cancer. 

But, the management of patients with massive PEs and an intracranial process can be challenging. Such patients are at risk for dying from shock or acute right heart failure. Therapies must be balanced against the risk of worsening or precipitating an intracranial bleed. A recent report with thrombolytic therapy in a patient with a PE and a brain tumor argues the <10% risk for hemorrhagic transformation must be balanced against the 25–100% case fatality rate for massive PEs [5]. However, there is little additional data to support this practice.

Another treatment option is catheter directed therapy—even in patients with contraindications to systemic thrombolytic therapy. These techniques involve direct mechanical fragmentation, aspiration, or direct thrombolytic therapy and have been shown to be effective and safe in patients with massive PE [6]. These techniques, when successful, are associated with a quick resolution of symptoms and hemodynamic instability can often be accomplished quickly. However, there is a lack of standardized protocols and no device or catheter has an approved indication by the United States Food and Drug Administration (FDA) for the treatment of pulmonary embolism. Likewise, no thrombolytic agent has FDA approval for direct pulmonary infusion. Nevertheless, recent guidelines describing the management of PE suggest that catheter-directed therapy can be an acceptable option for the treatment of massive PE and can potentially serve a life-saving role in an institutional management algorithm [2]. 

Cardiac surgery, because of presumed bleeding risks has been historically contraindicated in patients with severe intracranial pathology. Our recent experience [3] and Fukuda's report of 3 successful cases of salvage pulmonary embolectomy in patients with recent intracranial bleeding [7] contribute to the rationale of operating on these acutely ill patients—particularly in whom full anticoagulation with heparin for bypass might be theoretically less harmful than systemic lytic therapy.

Our case demonstrates the feasibility of extending the surgical management of massive PEs to patients with advanced brain cancers. While such interventions may be considered aggressive or futile in neurologically debilitated patients with known life-limiting medical problems, our experience suggests the contrary. Preoperative neuro-oncology consultation, projected a 6–12 months, albeit unpredictable, survival. In addition, this experience also emphasize the need for open communication, particularly with a Palliative Care team, and a reasonable set of expectations and advanced directives in patients with advancing diseases.

## 3. Conclusions

As experience with the surgical management of acute massive pulmonary embolism grows, indications can include patients traditionally considered at prohibitive risk or in whom thrombolytics are contraindicated. Our cases demonstrate that pulmonary embolectomy can be successful in patients with advanced brain tumors. Our experience also emphasizes that in each case, regardless of the comorbidities and management plan, that there needs to be careful consideration given to the patient's baseline and anticipated functional status, expected duration of survival, and most importantly, any known advances directives. In some centers, depending on team preferences and skills, catheter-based therapies may be an acceptable alternative to surgical options. While we advocate—based upon our experiences—surgical management, regardless of the preferred approach, an institutional algorithm for the treatment of massive PE should include patients who have contraindications to thrombolytic therapy.
